# Coexistence of hepatocellular carcinoma and focal nodular hyperplasia in a non-cirrhotic liver: A case report

**DOI:** 10.1016/j.ijscr.2025.111826

**Published:** 2025-08-18

**Authors:** Faten Limaiem, Rached Bayar, Mohamed Hajri, Aziz Atallah, Hafedh Mestiri

**Affiliations:** a University of Tunis El Manar, Faculty of Medicine of Tunis, Tunisia; b Pathology Department, Hospital Mongi Slim La Marsa, Tunisia; c Department of Visceral Surgery, Hospital Mongi Slim La Marsa, Tunisia

**Keywords:** Liver, Tumor, Hepatocellular carcinoma, Focal nodular hyperplasia, Case report

## Abstract

**Introduction and importance:**

The coexistence of hepatocellular carcinoma and focal nodular hyperplasia in a non-cirrhotic liver is rare and diagnostically challenging due to overlapping imaging features. This case report exposes key diagnostic pitfalls and stresses integrating clinical, imaging, and pathology data for accurate diagnosis and management.

**Case presentation:**

A 65-year-old woman with chronic hepatitis C and no cirrhosis presented with persistent generalized pruritus. Laboratory tests showed cholestatic liver abnormalities and an elevated alpha-fetoprotein level of 46.6 ng/mL. Computed tomography and magnetic resonance imaging suggested focal nodular hyperplasia, based on typical enhancement and a central scar. Due to discordance between imaging and tumor markers, she underwent resection of liver segment IVb with cholecystectomy. Histopathology revealed the unexpected coexistence of well-differentiated hepatocellular carcinoma adjacent to focal nodular hyperplasia.

**Clinical discussion:**

This case highlights the diagnostic challenge of differentiating focal nodular hyperplasia from hepatocellular carcinoma in non-cirrhotic patients. Although focal nodular hyperplasia usually shows distinctive imaging features, well-differentiated hepatocellular carcinoma can closely mimic them. When non-invasive methods are inconclusive, surgical resection may be needed for definitive diagnosis.

**Conclusions:**

Clinicians should maintain a high index of suspicion for hepatocellular carcinoma even in non-cirrhotic patients presenting with liver lesions suggestive of focal nodular hyperplasia and elevated tumor markers. This case highlights the importance of comprehensive evaluation and the role of surgery in ambiguous hepatic lesions.

## Highlights

• The coexistence of HCC and FNH is rare and challenging to diagnose.
• Elevated AFP > 20 ng/mL suggests malignancy, even with benign lesions.
• Histopathological examination is often needed for a definitive diagnosis.
• Surgical resection confirms diagnosis and provides treatment in ambiguous cases.
• High suspicion for HCC is needed for FNH-like lesions and elevated AFP.

## 1 Introduction

Focal nodular hyperplasia (FNH) is the second most common benign hepatic lesion, with a prevalence of 0.2–3 % in the general population [1]. It typically presents as a solitary, hypervascular mass, most often discovered incidentally in women of reproductive age. Multifocal FNH occurs in up to 20 % of cases and may coexist with other benign hepatic lesions, such as hemangiomas or hepatocellular adenomas [[1], [2], [3]]. FNH is widely regarded as a non-neoplastic, benign lesion with no proven risk of malignant transformation [[1], [2], [3]]. In contrast, hepatocellular carcinoma (HCC) is the most common primary malignant tumor of the liver, typically arising in cirrhotic livers due to chronic hepatitis B or C, alcohol-related liver disease, or nonalcoholic steatohepatitis. However, 10–20 % of cases occur in non-cirrhotic livers, particularly in association with viral hepatitis. The coexistence of FNH and HCC in non-cirrhotic livers is exceptionally rare and poses a diagnostic challenge, as HCC may mimic FNH on imaging [[4], [5], [6], [7], [8], [9], [10], [11]]. While isolated reports have described such coexistence including both conventional HCC and fibrolamellar variants the underlying pathogenic relationship remains unclear and poorly understood [[4], [5], [6], [7], [8], [9], [10], [11]].

In this paper, we report the seventh documented case of conventional HCC coexisting with FNH in a non-cirrhotic liver. This diagnostically challenging case involved a 65-year-old woman with chronic hepatitis C, whose liver lesion mimicked FNH on imaging but was found to harbor both lesions in the same segment. This report aims to raise awareness of this rare association, highlight potential diagnostic pitfalls, and underscore the importance of integrating clinical, serological, and pathological data to avoid misdiagnosis and guide appropriate management.

This case report adheres to the SCARE 2025 Criteria [12].

## 2 Case presentation

### 2.1 Patient history and presenting complaint

A 65-year-old woman with a significant past medical history of ischemic stroke (on aspirin prophylaxis), epilepsy (controlled with valproate), and anxiety-depressive disorder presented with persistent generalized pruritus as her chief complaint. She specifically denied concomitant jaundice, abdominal pain, or other gastrointestinal symptoms.

### 2.2 Physical examination

On presentation, the patient's vital signs were within normal limits, including a temperature of 37.2 °C, blood pressure of 125/78 mmHg, heart rate of 76 bpm, respiratory rate of 16 breaths/min, and oxygen saturation of 98 % on room air. The abdominal examination was unremarkable, with no tenderness, organomegaly, or palpable masses. Clinical examination revealed no signs of jaundice, hepatomegaly, or peripheral stigmata of chronic liver disease.

### 2.3 Diagnostic workup

**Fig. 1:**
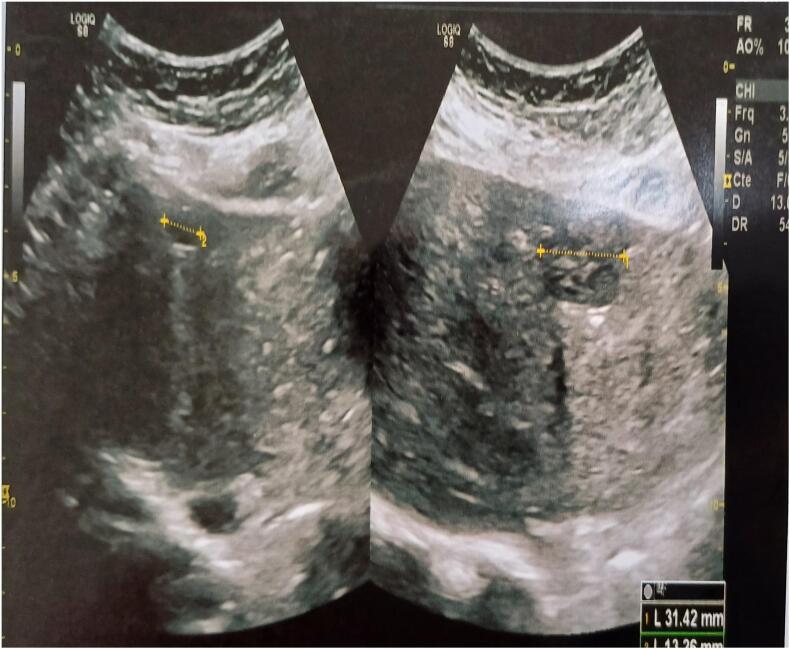
Abdominal ultrasound demonstrates two hypoechoic, heterogeneous nodular lesions with regular margins in a liver of normal volume. The larger lesion (31 mm) is located in segment IV, while the smaller (20 mm) is in segment VIII.

**Fig. 2:**
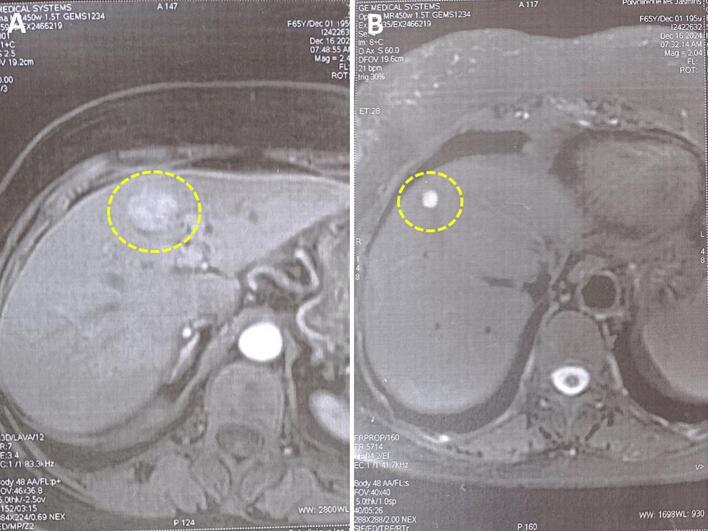
MRI findings of hepatic lesions. (A) Segment IVb shows a roughly rounded nodule with polycyclic margins, demonstrating subtle T2 hyperintensity and isointense T1 signal. (B) Segment IVa contains a non-enhancing 10-mm fluid-intensity lesion, characteristic of a simple biliary cyst.

The initial laboratory workup showed a cholestatic pattern, with alkaline phosphatase elevated to 320 IU/L (normal range: 40–129 IU/L) and gamma-glutamyl transferase increased to 185 IU/L (normal range: 8–61 IU/L). Total bilirubin was normal at 12 μmol/L (reference: <21 μmol/L), while transaminases were mildly elevated (ALT: 48 IU/L, reference: 7–55 IU/L; AST: 52 IU/L, reference: 8–48 IU/L). Serological testing confirmed chronic HCV infection, with positive anti-HCV antibodies and a high viral load of 850,000 IU/mL (undetectable threshold: <15 IU/mL). FibroTest assessment revealed minimal fibrosis (F0–F1 stage) and absent inflammatory activity (A0). A notably elevated alpha-fetoprotein level of 46.6 ng/mL (normal: <7 ng/mL) was observed. Abdominal ultrasound revealed a normally sized liver containing two hypoechoic, heterogeneous nodular lesions with regular margins (Fig. 1). Color Doppler demonstrated vascularization within these lesions, located in segment IV (31 mm) and segment VIII (20 mm). Contrast-enhanced computed tomography (CT) identified a 32 mm hypervascular lesion in segment IV, exhibiting persistent enhancement consistent with focal nodular hyperplasia (FNH). Subsequent MRI confirmed characteristic FNH features, including isointensity on T1-weighted sequences, mild hyperintensity on T2-weighted images, homogeneous arterial enhancement, and a central scar (Fig. 2A). The liver also demonstrated a 10-mm non-enhancing fluid-attenuated lesion in segment IVa, consistent with a biliary cyst (Fig. 2B).

### 2.4 Surgical management

Under general anesthesia, a right subcostal incision was performed to provide adequate exposure of the liver. The liver was mobilized, and intraoperative inspection confirmed the presence of a well-demarcated lesion in segment IVb. A cholecystectomy was carried out to facilitate access and visualization of the hepatic lesion. Vascular inflow to segment IVb was controlled using selective clamping of the Glissonian pedicle. Parenchymal transection was performed using an ultrasonic aspirator to minimize blood loss, with meticulous ligation of small vessels and bile ducts. The lesion was carefully resected with a sufficient margin of normal liver tissue to ensure complete removal. Hemostasis was secured, and the resected specimen was sent for histopathological examination. No intraoperative complications were encountered. The surgical field was irrigated, and a closed-suction drain was placed near the resection site. The incision was closed in layers, and the patient was transferred to the recovery unit for postoperative monitoring.

### 2.5 Pathological findings

**Fig. 3:**
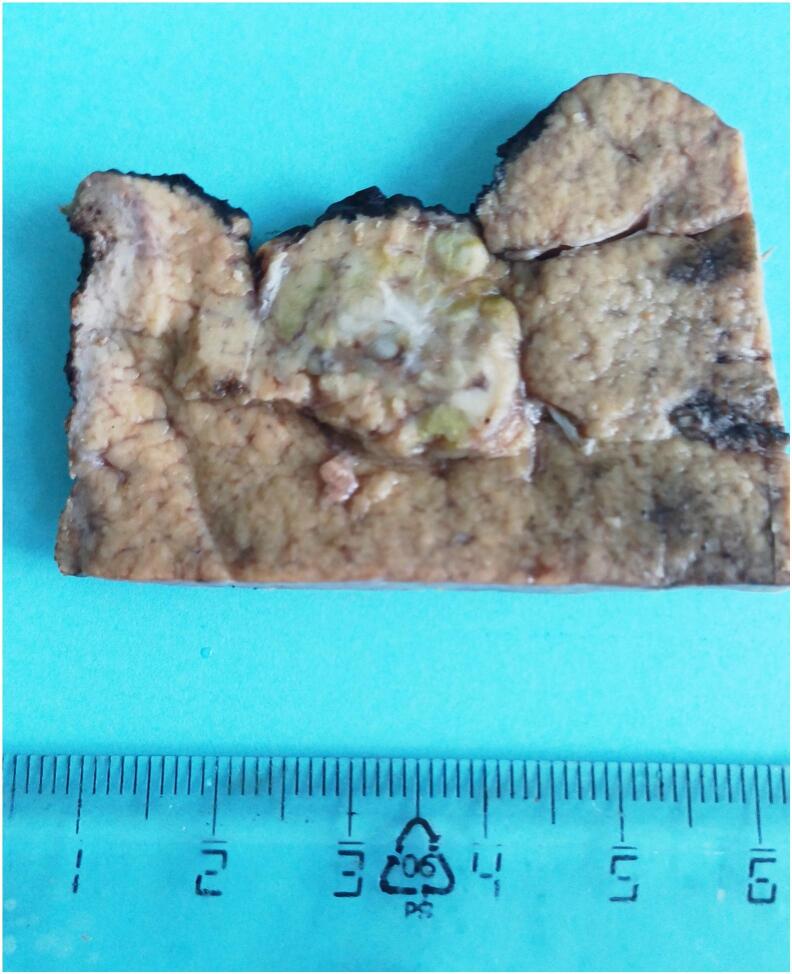
Gross examination of the resected liver specimen revealed a 30-mm nodule with polycyclic borders and a yellow-green cut surface, featuring a central stellate scar. (For interpretation of the references to colour in this figure legend, the reader is referred to the web version of this article.)

**Fig. 4:**
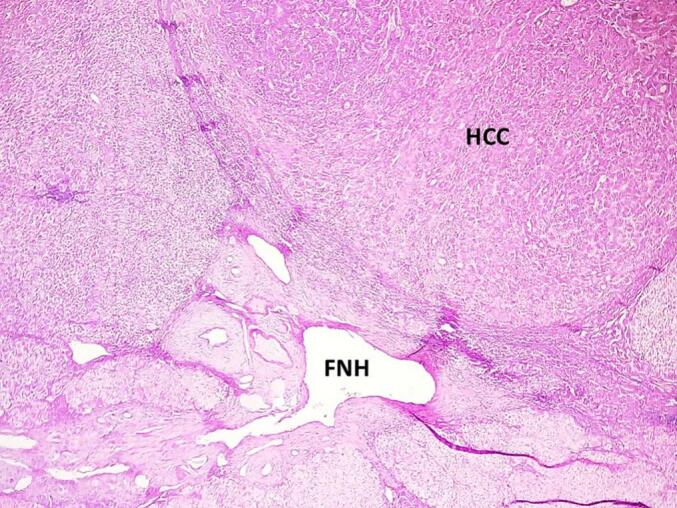
Histopathological examination demonstrates a central stellate scar composed of dense fibrous tissue with abnormally thick-walled blood vessels, consistent with focal nodular hyperplasia (FNH). Fibrous septa extend radially, delineating nodular architecture within the hepatic parenchyma. Adjacent to the scar, a well-demarcated nodule displays morphological features of well-differentiated hepatocellular carcinoma (HCC) (hematoxylin and eosin; original magnification ×40).

**Fig. 5:**
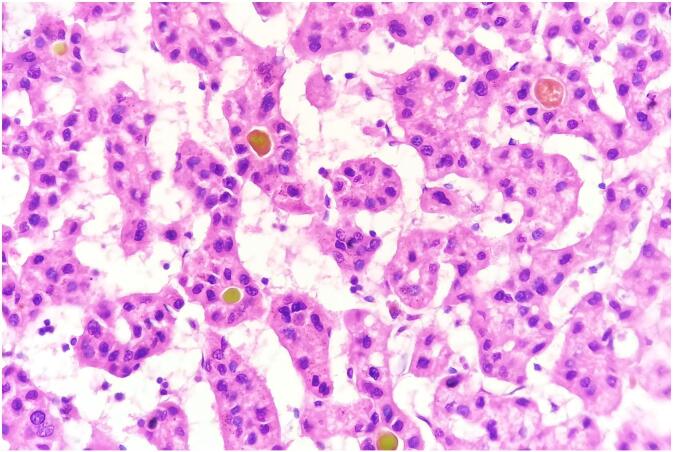
Well-differentiated hepatocellular carcinoma demonstrating characteristic trabecular and pseudoglandular growth patterns. Tumor cells show mild nuclear atypia and eosinophilic cytoplasm, with scattered extracellular bile pigment deposits (brownish-green granules) visible within pseudoglandular lumina (hematoxylin and eosin, magnification ×400). (For interpretation of the references to colour in this figure legend, the reader is referred to the web version of this article.)

The resected liver specimen (738 g; dimensions: 8.0 × 4.5 × 3.4 cm) contained a 30 mm nodule with a yellow-green cut surface and central stellate scarring (Fig. 3). Histopathological analysis confirmed the coexistence of well differentiated hepatocellular carcinoma (Edmondson-Steiner grade I) in proximity to an area of focal nodular hyperplasia (Fig. 4, Fig. 5).

### 2.6 Postoperative course and follow-up

The patient's postoperative recovery was uneventful, and she was discharged on postoperative day five. At her two-month follow-up, she remained asymptomatic, with no clinical or radiological evidence of disease recurrence.

## 3 Discussion

FNH is the second most common benign liver tumor, typically arising in women of reproductive age and lacking malignant potential. Its pathogenesis is generally attributed to localized vascular abnormalities, which trigger hepatocellular hyperplasia, polyclonal expansion, and fibrous tissue deposition [2]. HCC accounts for approximately 75 to 85 % of all primary liver cancers worldwide [8,9]. Most cases develop in cirrhotic livers, commonly due to chronic hepatitis B or C infection, alcoholic liver disease, or nonalcoholic steatohepatitis. However, 10 to 20 % of HCC arise in non-cirrhotic livers [8,9]. The coexistence of FNH and HCC in a non-cirrhotic liver is exceedingly rare. To date, only ten cases of HCC coexisting with FNH have been documented in the literature (Table 1) [[4], [5], [6], [7], [8], [9], [10], [11]], comprising 4 cases of FL-HCC and 6 cases of conventional HCC. Our case represents the seventh reported instance of conventional HCC with concurrent FNH, and the eleventh reported case overall when including all histological subtypes.

**Table 1:** Reported cases of concomitant focal nodular hyperplasia and hepatocellular carcinoma.

Author	Year	Age/sex	Tumor size	Treatment	Pathology	Evolution
Saul SH [4]	1987	NA	NA	NA	FL-HCC + FNH	NA
Davidson BR [5]	1990	16/M	6 cm	Partial hepatectomy of the quadrate lobe	FL-HCC + FNH	Tumor recurrence 7 years postoperatively
Muguti [6]	1992	3 cases	NA	NA	FL-HCC + FNH (n = 1)	NA
					HCC + FNH (n = 2)	
Saxena R [7]	1994	14/F	5 cm	Listed for transplantation	FL-HCC + FNH	Died (paracetamol overdose)
Chen TC [8]	2001	65/F	20 × 14 × 11 cm	Right hepatectomy	HCC + FNH	Not available
Coopersmith CM [9]	2002	43/F	7 × 6.5 × 3.5 cm	Right hemihepatectomy	HCC + diffuse FNH	Recurrence (6 cm mass); lost to follow-up
Cucchetti A [10]	2003	55/F	7 cm	Segments VI and VII resection	HCC + FNH	A 1 cm hepatic nodule was detected 6 months after surgery
Petsas T [11]	2006	23/F	2 masses, 9 cm and 4.5 cm	Resection of segments II and III	HCC + large FNH	Liver transplantation
						Patient alive (8 years of follow-up)
Our case	2025	65/F	3 cm	Segment IVb resection	HCC + FNH	No recurrence (2-month follow-up)

Abbreviations: FL-HCC: fibrolamellar hepatocellular carcinoma; FNH: focal nodular hyperplasia; HCC: hepatocellular carcinoma; NA: not available.

Histologically, the two lesions were adjacent but distinct, with no features suggesting that the HCC arose from the FNH. Given these observed associations between HCC and FNH, a potential pathogenetic link has been hypothesized. However, molecular investigations into tumor clonality have produced inconsistent findings. Gaffey et al. [14] and Paradis et al. [15] analyzed X-chromosome inactivation patterns in FNH and reached opposite conclusions—one suggesting monoclonality, the other polyclonality. From a pathophysiological standpoint, the synchronous occurrence of FNH and HCC remains puzzling. FNH is thought to result from a hyperplastic response to vascular anomalies, while HCC develops through pathways involving chronic inflammation, oxidative stress, and genomic instability. In our patient, chronic hepatitis C infection was present, but liver fibrosis was minimal (F0–F1). This raises the possibility that persistent viral inflammation and altered vascular architecture could contribute to tumorigenesis, even in the absence of cirrhosis. Similar observations were made by Saxena et al. [7], who suggested that hypervascularity in fibrolamellar HCC may induce reactive parenchymal hyperplasia resembling FNH. Radiologically, differentiating FNH from well-differentiated HCC is challenging due to overlapping features such as arterial phase hyperenhancement and the presence of a central scar. Hepatobiliary-specific contrast agents can enhance diagnostic accuracy, but well-differentiated HCC may still exhibit contrast retention, mimicking FNH [3,16,17]. In our patient, typical imaging features of FNH were observed; however, an elevated AFP level raised concern for malignancy. According to EASL guidelines, AFP values above 20 ng/mL warrant further evaluation for HCC, regardless of reassuring imaging findings [3,[16], [17], [18]].

In several cases, including ours, preoperative biopsy was either not performed or proved inconclusive due to sampling limitations, often capturing only benign tissue while missing coexistent malignancy. This underscores the role of surgical resection as the definitive approach when clinical, radiological, and serological findings are discordant, offering both diagnostic clarity and therapeutic benefit. AFP remains a key biomarker in the diagnosis, surveillance, and follow-up of HCC [18]. In our case, surgery was pursued without prior histological confirmation due to a significant mismatch between the elevated AFP level (46.6 ng/mL) and imaging suggestive of focal nodular hyperplasia. Fine-needle aspiration was deemed inadequate, given its limitations in detecting coexisting malignancy and characterizing complex lesions, making resection essential for accurate diagnosis and effective treatment. While mild AFP elevations (typically <100 ng/mL) can occur in non-malignant conditions such as cirrhosis, chronic hepatitis, pregnancy, or germ cell tumors [18] AFP levels are usually normal in FNH. Rare cases of FNH associated with AFP levels between 40 and 60 ng/mL have been described, potentially linked to regenerative activity or progenitor cell proliferation within the lesion [3,18]. Thus, AFP assessment is strongly recommended in all newly identified liver lesions. A markedly elevated or rising AFP level, even in the presence of benign imaging, should prompt further evaluation for malignancy. This case report offers a valuable contribution by documenting the rare coexistence of HCC and FNH in a non-cirrhotic liver, supported by comprehensive radiologic–pathologic correlation and surgical confirmation. A key strength lies in the detailed multidisciplinary evaluation that underscores the diagnostic pitfalls when imaging and serologic markers diverge. However, the absence of molecular or genetic clonality analysis limits further insight into potential pathogenetic links between the two lesions.

## 4 Conclusion

In conclusion, this rare case of synchronous hepatocellular carcinoma and focal nodular hyperplasia in a non-cirrhotic liver with chronic hepatitis C infection highlights the diagnostic complexity posed by overlapping imaging features and atypical clinical profiles. The discordance between benign-appearing radiologic findings and elevated alpha-fetoprotein levels illustrates how well-differentiated HCC can closely mimic FNH, increasing the risk of misdiagnosis. This case emphasizes the importance of maintaining clinical vigilance and adopting a multidisciplinary diagnostic strategy that integrates imaging, tumor markers, and histopathological confirmation. When noninvasive evaluations are inconclusive, surgical resection remains essential for both definitive diagnosis and treatment. Finally, this report underscores the need to refine diagnostic algorithms and develop molecular or vascular biomarkers to improve preoperative differentiation between benign and malignant hepatic lesions especially in non-cirrhotic patients with chronic viral hepatitis who fall outside conventional risk categories.

## Consent statement

Written informed consent was obtained from the patient for publication of this case report and accompanying images. A copy of the written consent is available for review by the Editor-in-Chief of this journal on request.

## Consent

Written informed consent was obtained from the patient for publication of this case report and accompanying images. A copy of the written consent is available for review by the Editor-in-Chief of this journal on request.

## Ethical approval

The hospital's ethics committee has granted an exemption for ethical approval for the case report. Ethical approval is unnecessary for case reports that are not classified as research within our institution.

## Ethical approval

The ethics committee of Mongi Slim University Hospital in Marsa, Tunisia, has granted an exemption for ethical approval for the case report. Ethical approval is unnecessary for case reports that are not classified as research within our institution.

## Guarantor

Dr. Faten Limaiem.

## Provenance and peer review

Not commissioned, externally peer-reviewed.

## Sources of funding

This research did not receive any specific grant from funding agencies in the public, commercial, or not-for-profit sectors.

## Funding

This research did not receive any specific grant from funding agencies in the public, commercial, or not-for-profit sectors.

## Author contribution

Dr. Faten Limaiem prepared and finalized the histopathological figures, performed critical manuscript review, and provided substantial intellectual contributions. Dr. Mohamed Hajri, Prof. Rached Bayar, and Prof. Hafedh Mestiri contributed to the study conception, design, data acquisition, and analysis. Dr. Aziz Atallah and Dr. Mohamed Hajri provided expert guidance during the research process, participated in manuscript revision, and approved the final version for submission. All authors critically reviewed the manuscript and approved its final version, ensuring academic integrity and accuracy.

## Declaration of competing interest

None declared.
